# The OceanDNA MAG catalog contains over 50,000 prokaryotic genomes originated from various marine environments

**DOI:** 10.1038/s41597-022-01392-5

**Published:** 2022-06-17

**Authors:** Yosuke Nishimura, Susumu Yoshizawa

**Affiliations:** 1 Atmosphere and Ocean Research Institute, The University of Tokyo, Chiba, 277-8564 Japan; 2 Graduate School of Frontier Sciences, The University of Tokyo, Chiba, 277-8563 Japan; 3 Collaborative Research Institute for Innovative Microbiology, The University of Tokyo, Tokyo, 113-8657 Japan; 4 Present Address: Research Center for Bioscience and Nanoscience (CeBN), Research Institute for Marine Resources Utilization, Japan Agency for Marine-Earth Science and Technology (JAMSTEC), Yokosuka, Kanagawa 237–0061 Japan

**Keywords:** Environmental microbiology, Microbial communities

## Abstract

Marine microorganisms are immensely diverse and play fundamental roles in global geochemical cycling. Recent metagenome-assembled genome studies, with particular attention to large-scale projects such as *Tara* Oceans, have expanded the genomic repertoire of marine microorganisms. However, published marine metagenome data is still underexplored. We collected 2,057 marine metagenomes covering various marine environments and developed a new genome reconstruction pipeline. We reconstructed 52,325 qualified genomes composed of 8,466 prokaryotic species-level clusters spanning 59 phyla, including genomes from the deep-sea characterized as deeper than 1,000 m (n = 3,337), low-oxygen zones of <90 μmol O2 per kg water (n = 7,884), and polar regions (n = 7,752). Novelty evaluation using a genome taxonomy database shows that 6,256 species (73.9%) are novel and include genomes of high taxonomic novelty, such as new class candidates. These genomes collectively expanded the known phylogenetic diversity of marine prokaryotes by 34.2%, and the species representatives cover 26.5–42.0% of prokaryote-enriched metagenomes. Thoroughly leveraging accumulated metagenomic data, this genome resource, named the OceanDNA MAG catalog, illuminates uncharacterized marine microbial ‘dark matter’ lineages.

## Abstract (summary)

**Table:** 

Measurement(s)	microbial community
Technology Type(s)	marine metagenome
Sample Characteristic - Organism	Bacteria • Archaea
Sample Characteristic - Environment	marine biome

## Background & Summary

Marine microorganisms have shaped Earth’s environment and played crucial roles in controlling the global climate^1,2^. Genome-based knowledge is essential to understand microorganisms in various aspects, including their phylogeny, evolution, metabolism, and physiology. Though difficulty in isolation has limited the genome-based knowledge of marine microorganisms, the success of culture-independent genome reconstruction techniques such as metagenome-assembled genomes (MAGs) and single-amplified genomes (SAGs) have changed our understanding of microbial ecosystems. Genome information of marine microorganisms supplied by these approaches enabled the uncovering of new lineages identified as participants in crucial biogeochemical cycling (e.g., nitrogen fixation^3^ and carbon fixation^4,5^), the characterization of metabolic potentials of uncultured lineages^6–10^, and the reconstruction of deep evolutionary trajectories of microorganisms^11,12^.

Metagenomes of *Tara* Oceans Expeditions^13,14^ have been repeatedly subjected for genome reconstruction^3,4,10,11,15–17^. In contrast, large-scale metagenome data from which relatively little effort for genome reconstruction (e.g., metagenomes of GEOTRACES^18^, Station ALOHA^19^, Saanich Inlet^20^) or from which genomes of limited taxa were reported (e.g., metagenomes of the Canada Basin^21^) has been published. Moreover, genome reconstruction methodologies in many previous studies are considered inefficient (e.g., use of a single binning algorithm and coverage profile limited to a single or a few samples^22^). Genome reconstruction using an improved methodology and applying it to a large-scale metagenome dataset is thus promising for expanding our genomic knowledge of marine microorganisms.

**Fig. 1:**
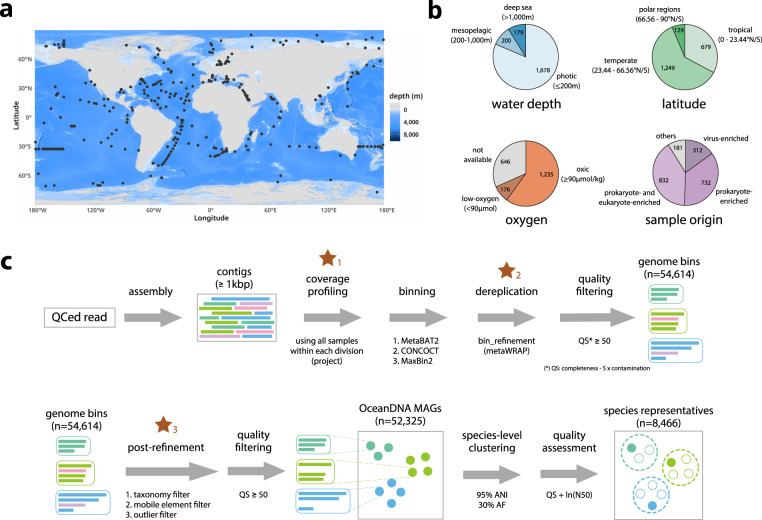
Overview of the study. (**a**) Geographic distribution of the 2,057 metagenomes analyzed in this study (shown by black points). The map was drawn using marmap^77^ and ggplot2 (https://ggplot2.tidyverse.org/). (**b**) Origin of the metagenome samples. Details of the sample origin were described in the main text. (**c**) Schematic representation of the pipeline for MAG reconstruction. Three key processes were highlighted with brown stars. Source data is available in Supplementary File S1.

**Fig. 2:**
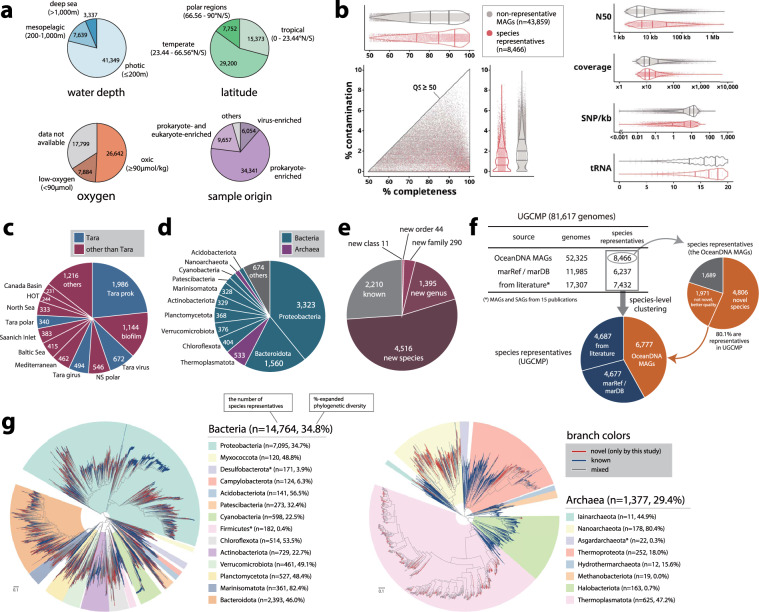
Origin, quality, and novelty of the OceanDNA MAGs. (**a**) Origin of the OceanDNA MAGs. Types of the fraction were described in the main text. (**b**) Genome statistics for species representatives and non-representatives. Lines in violin plots indicate quartiles that were estimated based on density profiles. (**c**) Origin of metagenome divisions of the 8,466 species representatives. (**d**) Phyla of the species representatives assigned by GTDB-Tk. (**e**) The potential taxonomic novelty of the species representatives assessed using GTDB-Tk. (**f**) Origins and compositions of the unified catalog UGCMP and the species representatives. (**g**) Bacterial (left) and archaeal (right) phylogenetic trees of the species representatives of UGCMP. The trees were midpoint rooted for visualization purposes. The number of species representatives and %-expanded phylogenetic diversity was described for individual phyla, of which the number of species was at least 100 for bacteria and 10 for archaea. These phyla were highlighted in the trees with the corresponding colors. If a phylum was not monophyletic in the trees, only the largest monophyletic unit was highlighted (three phyla represented by asterisks in the legend). Note that %-expanded phylogenetic diversity was estimated using all the genomes of UGCMP (not limited to the species representatives). Source data is available in Supplementary File S3.

We aimed to build a comprehensive genome catalog of marine prokaryotes by taking advantage of accumulated metagenomic data. Practically, two methodological focuses of this study were defined as (1) to compose a large-scale metagenome dataset that covers diverse marine environments including less explored regions such as deep-sea, low-oxygen zones, and polar regions and (2) to develop a new genome reconstruction pipeline to maximize the quality of reconstructed genomes. Here, we collected 2,057 published metagenomes (>29 Tera bps of sequences) originating from diverse marine environments (Fig. 1a,b), primarily focused on water samples (n = 1,890). In addition, samples of sediment traps^23,24^ (n = 63) and biofilms^25^ (n = 104) were included. Then, to improve the quality of genomes, we developed a genome reconstruction pipeline that includes three key processes (Fig. 1c). As a result, we reconstructed 52,325 qualified prokaryotic genomes that were QS (quality score: %-completeness - 5 x %-contamination) ≥50, named the OceanDNA MAGs. These genomes were reconstructed from various marine environments, including genomes originated from deep-sea regions deeper than 1,000 m (n = 3,337; from 179 metagenomes), low-oxygen zones of <90 μmol O_2_ per kg water (n = 7,884; from 176 metagenomes), and polar regions (n = 7,752; from 129 metagenomes) (Fig. 2a).

The OceanDNA MAGs were composed of 8,466 species-level clusters. Genomes were identified as species representatives if the genome quality was the best within each species-cluster (assessed by ‘QS + ln(N50)’). The median genome completeness and contamination of the OceanDNA MAGs were estimated as >80% and <2%, respectively (Fig. 2b). The species representatives were derived from various metagenomic projects (divisions) and not dominated by ones from *Tara* Oceans (Fig. 2c). Taxonomic classification based on the genome taxonomy database (GTDB) release 05-RS95^26^ showed that the OceanDNA MAGs covered various marine prokaryotic lineages spanning 59 phyla (Fig. 2d). According to the classification, 11 species representatives were not assigned to any existing class, suggesting that these species potentially belong to new classes. Likewise, we identified 44 species of new orders, 290 new families, and 1,395 new genera (Fig. 2e). Overall, most representatives (n = 6,256; 73.9%) were not assigned to existing species in the database.

**Fig. 3:**
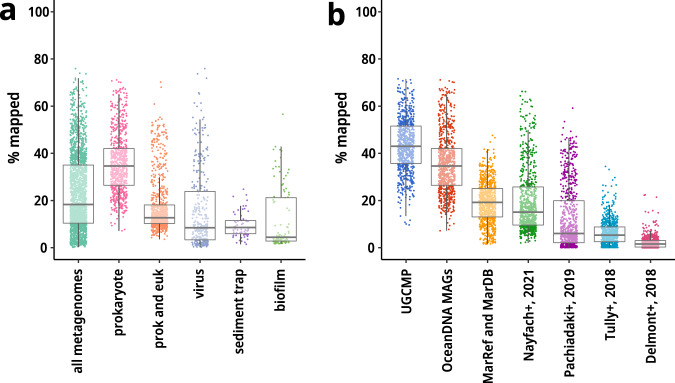
Recruitment of metagenomic reads. The fraction of mapped reads of 2,057 metagenomes was evaluated at ≥95% nucleotide identity. (**a**) Recruitment onto the species representatives of the OceanDNA MAGs. The x-axis shows types of metagenome sources. prokaryote: prokaryote-enriched metagenomes, prok and euk: prokaryote- and eukaryote-enriched metagenomes, virus: virus-enriched metagenomes. (**b**) Recruitment of prokaryote-enriched metagenome reads. The x-axis shows genome collections. Note that all these genome collections include only species representatives of qualified genomes (i.e., QS ≥ 50). UGCMP and OceanDNA MAGs include genomes reconstructed in this study. Nayfach+, 2021^66^, Pachiadaki+, 2019^5^, Tully+, 2018^16^, and Delmont+, 2018^3^ are reported genome collections. For Nayfach+, 2021, genomes are limited to the ones that ‘ecosystem type’ is marine. Source data is available in Supplementary File S1.

The novelty of the OceanDNA MAGs was further evaluated using published marine prokaryotic genomes (n = 29,292). Among the 8,466 species representatives, 80.1% was not overlapped with the published genomes at the species level (56.8%) or was overlapped but of superior genome quality (assessed by ‘QS + ln(N50)’) to the published genomes (23.3%) (Fig. 2f). The OceanDNA MAGs expanded the known phylogenetic diversity of marine prokaryotes by 34.2%, evaluated by the sum of branch length of bacterial/archaeal phylogenomic trees (Fig. 2g). The species representative genomes collectively covered 26.5–42.0% of metagenomic reads of prokaryote-enriched metagenomes at ≥95% nucleotide identity (Fig. 3a). The OceanDNA MAG catalog is available as an unprecedented-scale genome resource of marine prokaryotes that facilitates characterization of microbial ‘dark matter’ lineages and elucidation of yet unsolved questions of marine microbial ecosystems.

## Methods

### Collection of metagenomes

We composed a dataset of marine metagenomes derived from a broad range of geographic regions (Fig. 1a). Various research groups published these metagenomes, and we organized these into 24 divisions for operational purposes, considering various factors such as related publications, research groups, and geographic regions (Table 1). These metagenome samples include ones collected from long-distance cruises (e.g., *Tara* Oceans^27–29^, GEOTRACES^18^, and Malaspina^30^) and from time-series or transect sampling in a specific marine region (e.g., the Mediterranean Sea^31,32^, the Baltic Sea^33^, the Saanich Inlet^20^, Station ALOHA^19^, and the San Pedro Channel^34^). The metagenome dataset was focused on water samples (n = 1,890; 91.9% of collected samples), but metagenomes derived from sediment traps^23,24^ (n = 63) and *in situ* formation of biofilms^25^ (n = 104) were also included. Associated metadata such as location, date, depth, oxygen concentration was collected from the original publication and the BioSample database (Supplementary File S1). The metagenomic samples were derived from pole-to-pole (76.96°S–85.02°N), sea surface to deep-sea (0–10,899 m below sea level), oxic to anoxic zones, and coastal to pelagic seas (Fig. 1a,b). The samples contain ones from aphotic zones (179 metagenomes from deeper than 1,000 m; 200 metagenomes from 200–1,000 m), low-oxygen zones (73 dysoxic (20–90 μmol/kg), 86 suboxic (1–20 μmol/kg), and 17 anoxic (<1 μmol/kg) metagenomes, according to ref. ^35^ Fig. 1b). Most water samples were originated from prokaryote-enriched fractions (water pass through a prefilter of 0.45–5 µm pore and collected on a filter of 0.1–0.45 µm pore; n = 732), prokaryote- and eukaryote-enriched fractions (pass through a prefilter of 20 µm pore or no prefilter and collected on a filter of 0.2–0.8 µm pore; n = 832), or virus-enriched fractions (pass through a prefilter of 0.2–0.22 µm pore; n = 312; Fig. 1b). Overall, these metagenomes cover various marine environments.

**Table 1:** 24 metagenome divisions.

division name	related publication (selected)	samples	QCed read (Gbp)	MAGs
*Tara* prok	Sunagawa *et al*.^27^	139	4,935	8,624
Saanich Inlet	Hawley *et al*.^20^	85	1,041	5,087
NS polar	Cao *et al*.^62^	59	847	3,511
*Tara* virus	Gregory *et al*.^28^	131	3,887	3,271
Monterey bloom	Nowinski *et al*.^44^	84	681	3,223
biofilm	Zhang *et al*.^25^	130	2,577	3,209
GEOTRACES	Biller *et al*.^18^	610	4,998	3,063
North Sea	Kruger *et al*.^60^	38	832	3,019
*Tara* polar	Salazar *et al*.^29^	41	1,416	2,762
*Tara* girus	Sunagawa *et al*.^27^	59	1,612	2,757
Baltic Sea	Alneberg *et al*.^33^	81	566	2,335
Mediterranean	Lopez-Perez *et al*.^78^	37	599	2,292
	Haro-Moreno *et al*.^79^			
	Martin-Cuadrado *et al*.^80^			
HOT	Mende *et al*.^19^	85	1,000	2,109
Malaspina	Acinas *et al*.^30^	72	209	1,320
	Gregory *et al*.^28^			
Med. coastal	Galand *et al*.^32^	40	276	1,243
Canada Basin	Colatriano *et al*.^21^	12	362	1,083
Hawaii bloom	Wilson *et al*.^81^	88	530	641
San Pedro Channel	Sieradzki *et al*.^34^	65	1,527	554
	Ignacio-Espinoza *et al*.^82^			
sediment trap	Poff *et al*.^24^	63	470	506
low oxygen	Thrash *et al*.^6^	26	123	476
	Tsementzi *et al*.^83^			
	Glass *et al*.^84^			
Atlantic	Bergauer *et al*.^85^	7	180	451
Red Sea	Haroon *et al*.^86^	45	83	319
NW Pacific	Saw *et al*.^10^, Li *et al*.^87^	35	96	248
Baltic Sea virus	Nilsson *et al*.^88^	25	261	222
total		2,057	29,110	52,325

### Sequence assemblies and metagenome binning

We downloaded metagenomic sequence data in a paired-end layout from NCBI SRA and quality controlled using Trimmomatic^36^ v0.35, with ‘LEADING:20 TRAILING:20 MINLEN:60’. If one side of the pair was discarded due to its low quality, the other was retained when it passed the quality control. The quality-controlled reads were assembled in a sample-by-sample manner (i.e., all the quality-controlled reads from one sample were used in one assembly) using MEGAHIT^37^ v1.1.4. We retained resulting contigs of ≥1 kbps. Sequence read and assembly statistics were shown in Supplementary File S1.

We then calculated a coverage profile of metagenomic contigs using all metagenomes belonging to the same division for better binning performance (Table 1; see also ‘Technical Validation’). An exception was applied to the division of GEOTRACES, which includes many metagenomes (n = 610). This division was split into six subdivisions, and the coverage profiles were calculated within each subdivision (Supplementary File S1). Read mapping was performed by bowtie2^38^ v2.3.5.1 using the quality-controlled paired-end reads. The mapping result was sorted by samtools (http://www.htslib.org/) v1.9, and coverage was calculated by jgi_summarize_bam_contig_depths that is bundled in MetaBAT2^39^, customizing a parameter ‘–percentIdentity’ set to 90. We then performed metagenome binning using three algorithms, MetaBAT2^39^ v2.12.1, MaxBin2^40^ v2.2.6, and CONCOCT^41^ v1.0.0. These algorithms were run with default settings, but for MetaBAT2, the ‘–minContig’ parameter was set to 1,500 following the software instruction, which states this value should not be less than 1,500. The resulting bins were then dereplicated and merged using the bin_refinement module of MetaWRAP^42^ v1.2.1, with minimum completion set to 50. The quality score (QS) was defined as ‘%-completeness - 5 x %-contamination’, and genomes of QS ≥ 50 were retained. Completeness and contamination of genome bins were estimated by taxon-specific sets of single-copy marker genes through the lineage-specific workflow of CheckM v1.0.13^43^. After removal of genomes likely derived from an internal standard (n = 63; *Thermus thermophilus* and *Blautias producta*^44^), 54,614 genome bins were obtained (Fig. 1c).

### Post-refinement of genome bins

For quality improvement of the reconstructed genome bins, we developed a post-refinement module to decontaminate potential misassigned contigs for each genome bin (Fig. 1c; see also ‘Technical Validation’). This module consists of three independent decontamination filters: (1) taxonomic filter, (2) mobile element filter, and (3) outlier filter. First, the taxonomic filter was designed to detect taxonomically inconsistent contigs with each genome. Coding regions were predicted with prodigal^45^ v2.6.3, and resulting proteins were used as input of CAT and BAT^46^ v5.0.3 to assign taxonomy for contigs and genomes, respectively. CAT and BAT were run with the default setting using NCBI Taxonomy downloaded in January 2020. Then, predicted taxonomy was quality controlled to remove the less reliable assignment. Namely, predicted taxonomy was recursively trimmed from the low level until either of the following three types of assignment are not detected:

A) ‘Suggestive’ taxonomic assignment that is less confident, indicated by stars in the BAT and CAT output
B) Very low-level assignment equal to or lower than species-level
C) Some ambiguous assignments (i.e., classified as ‘environmental samples’ or classifications start with ‘unclassified’).

A pair of a genome and its contig was taxonomically consistent only if the lowest common ancestor of the genome and the contig was the same as either of them. For example, suppose taxonomy of a genome is ‘class C1; order O1; family F1’, a contig is taxonomically consistent if taxonomy of the contig is like ‘class C1; order O1’ or ‘class C1; order O1; family F1; genus G1’, and inconsistent if it is like ‘class C1; order O1; family F2’ or ‘class C1; order O2.’

Second, the mobile element filter was designed to remove possible contamination of viral and plasmid contigs within genome bins. As genome bins are likely contaminated with viral and plasmid contigs that have similar coverage and nucleotide composition to the genome^22^, although these contigs might be actual parts of the genome as a provirus and a plasmid, we adopted a conservative approach that removes possible mobile elements. First, circular contigs were identified as potential viral and plasmid contigs by detecting terminal redundancy through ccfind^47^ (https://github.com/yosuken/ccfind). Second, viral contigs were detected using additional two types of methods. VirSorter^48^ v1.0.6 was used to detect viral contigs of ≥3 kb. The prediction result of category 1–6 was considered viral, but for category 4–6 (predicted as provirus), only if the length of the viral region was ≥50% of the total length, the contig was considered as viral. To supplement the detective power for short contigs (1 kb to 10 kb), we additionally scanned for *terL* genes that are one of the hallmark genes of prokaryotic viruses by following steps. We prepared 11 *terL* HMMs (Supplementary File S2) constructed from *terL* protein sequences obtained from previously identified aquatic viral MAGs (EVGs: circularly assembled environmental viral genomes)^47^. We searched for *terL* candidates using hmmsearch (HMMER^49^ v3.2.1; evalue <1e-10) with the 11 HMMs as queries. We validated sequence homology of the candidates with known *terL* genes using pipeline_for_high_sensitive_domain_search (https://github.com/yosuken/pipeline_for_high_sensitive_domain_search), which utilizes jackhmmer (HMMER^49^ v3.2.1) to build a protein HMM of each gene and HHsearch^50^ (HH-suite^51^ v3.2.0) to identify homology between the built HMMs and *terL* HMMs included in pfam 32.0. The candidates were identified as *terL* if the best hit is one of the *terL* domains (i.e., Terminase_1, Terminase_3, Terminase_6, Terminase_GpA, DNA_pack_N, Terminase_3C, and Terminase_6C) among all the pfam domains and if the probability of the HHsearch hit is >97%. We used proteins encoded in EVGs as a database of jackhmmer (jackhmmer parameters: ‘-N 5 --incE 0.001 --incdomE 0.001’).

Third, the outlier filter was designed to detect outlier contigs in coverage and tetranucleotide frequency (<−2.5 or >2.5 s.d. within each genome bin). Principal component analysis was performed using the prcomp function of R v3.6.2 (with default parameters), and the first primary component was evaluated. As a coverage profile, a part (related to contigs of the bin) of a coverage profile used for binning was extracted and normalized within each sample. Contigs identified as outliers were removed from the genome bin. Overall, after detecting and removing possible contamination using these three filters, completeness and contamination of each genome bin were again estimated with the lineage-specific workflow of CheckM.

Finally, 52,325 genomes of QS ≥ 50 were obtained and named the OceanDNA MAGs^52,53^ (Table S2). The OceanDNA MAGs reconstructed from various marine environments and size-fractions (Fig. 2a), including deep-sea deeper than 1,000 m (3,337 genomes from 176 samples), low-oxygen zones of <90 μmol O_2_ per kg water (7,884 genomes from 176 samples), polar regions (7,752 genomes from 129 samples), viral enriched fractions (pass through a filter of 0.2 or 0.22 µm pore; 5,998 genomes from 312 samples). Basic statistics of the genomes (e.g., total length and N50 of the assembly) were summarized using QUAST^54^ v5.0.2 (Supplementary File S3). Ribosomal RNAs (rRNAs) and transfer RNAs (tRNAs) were identified using Barrnap v0.9 (https://github.com/tseemann/Barrnap) and tRNAscan-SE^55^ v2.0.5, respectively. The identified rRNAs include the complete sequences and >25% fragments of the whole length. Read coverage and degree of heterogeneity of the genomes were assessed as follows. Metagenomic reads were back mapped with bowtie2^38^ v2.3.5.1 with the default setting using quality-controlled paired-end reads of a metagenome from which each genome was derived. The mapping result was sorted using samtools (http://www.htslib.org/) v1.9. Mappings of ≥95% identity, ≥80 bp, and ≥80% aligned fraction of the read length were extracted using msamtools (https://github.com/arumugamlab/msamtools) that are bundled in MOCAT2^56^ v2.1.3. The mean read coverage was calculated using the samtools sub-command ‘depth’. SNP site identification was performed only on sites of which the read coverage was at least 10. SNP sites were identified if the proportion of the dominant nucleotide, calculated using the samtools sub-command ‘mpileup’, was no more than 0.8. The degree of heterogeneity was evaluated by the proportion of SNP sites to all tested sites.

### Taxonomic assignment and their novelty evaluation using GTDB

We performed species-level clustering and identified species representatives of the OceanDNA MAGs through the following two rounds. First, for each of the 24 divisions, species-level clustering was performed using dRep^57^ v2.2.2 with a cutoff value of average nucleotide identity set as 95% and aligned fraction as 30%. We identified genomes of species representatives if ‘QS + ln(N50)’ was the highest within each species-level cluster. From the 24 divisions, 13,357 species representatives were identified at this round. Then, the secondary clustering was performed among these representatives using dRep, and 8,466 species-level clusters were obtained. The representatives of the species-level clusters were identified using the same criteria. The median genome completeness and contamination of both the species representatives (n = 8,466) and non-representatives (n = 43,859) were estimated as >80% and <2%, respectively (Fig. 2b). The species representatives showed higher completeness than non-representatives (85.09% and 80.66%, the median values), lower contamination (1.18% and 1.93%), larger N50 (11.6 kb and 6.2 kb), similar read coverage (12.87 and 12.91), a lower degree of polymorphism (3.97 and 7.94 SNP sites per kb), more unique tRNAs included (17 and 16), and a similar proportion of genomes with 16S rRNA (6.67% and 6.79%). We underline that the species representatives were originated from various metagenomic projects and not dominated by ones from *Tara* Oceans (Fig. 2c).

The OceanDNA MAGs were taxonomically classified using GTDB (Genome Taxonomy DataBase) release 05-RS95^26^ through the classify workflow of GTDB-Tk^58^ v1.3.0. As the classification based on GTDB, the species representatives spanned 59 phyla (Fig. 2d). Of these, 11 species representatives were not assigned to any existing class, suggesting that these species potentially belong to new classes. Likewise, it was suggested that 44 species representatives belong to new orders, 290 belong to new families, 1,395 belong to new genera, and 4,516 belong to new species (Fig. 2e). Overall, most species representatives (n = 6,256; 73.9%) were not assigned to existing species in the database.

### Novelty evaluation using published marine genomes

We comprehensively collected published genomes of marine prokaryotes for further novelty assessment of the OceanDNA MAGs. First, genomes in MarDB and MarRef^59^ v5.0, curated genome collections of marine prokaryotes derived from isolates/SAGs/MAGs, were downloaded (n = 14,209). Second, to supplement these with recently published genomes or genomes not stored in NCBI, we collected genomes (n = 26,946; SAGs and MAGs) of marine origin from 15 research articles^3,5,6,10,23,25,29,60–67^ (Supplementary File S4). After selection of qualified genomes (QS ≥ 50), 29,292 genomes were retained in total (11,985 from marRef/MarDB and 17,307 genomes from the 15 articles; Supplementary File S5). We then organized a unified genome catalog of marine prokaryotes (UGCMP; n = 81,617), composed of the 29,292 published genomes and the 52,325 OceanDNA MAGs (Fig. 2f). We identified species representatives of UGCMP by following two steps. Species-level clusters (n = 13,669) and the representatives were identified separately for MarDB/MarRef and each publication, using the same criteria as the OceanDNA MAGs. After unifying the species representatives of OceanDNA MAGs (n = 8,466) and published marine genomes (n = 13,669) into one set, the second-round species-level clustering was performed with the same conditions. We finally identified 16,141 species representatives of UGCMP using the same criteria (Supplementary File S6). The OceanDNA MAGs exclusively composed 4,806 species-level clusters (56.8% of the species representatives of the OceanDNA MAGs) and were selected as species representatives in 1,971 non-exclusive species-level clusters (23.3% of the species representatives of OceanDNA MAGs), showing the best genome quality (regarding ‘QS + ln(N50)’) among each cluster. Overall, a large part (80.1%; n = 6,777) of the species representatives of the OceanDNA MAGs was still species representatives in UGCMP.

We then assessed phylogenomic diversity of UGCMP for bacteria (n = 74,214) and archaea (n = 7,403). For domain and phylum-level classification, taxonomic assignment of UGCMP genomes was performed using GTDB release 05-RS95 and GTDB-Tk v1.3. Phylogenomic trees of bacteria and archaea were reconstructed with FastTree v2.1.11 (option: ‘-wag -gamma’) using alignments built by GTDB-Tk (Fig. 2g). The alignments included 5,040 sites of high phylogenetic signal from 120 single-copy marker genes for bacteria and 5,124 sites from 122 genes for archaea. After midpoint rooting using gotree (https://github.com/evolbioinfo/gotree) v0.4.0, a sum of branch length was calculated for two categories: (1) branches that were represented only by the OceanDNA MAGs (2) branches that were other than (1). The expanded phylogenetic diversity by the OceanDNA MAGs was 34.2% (34.8% for bacteria and 29.4% for archaea), estimated from a ratio of (1) to (2).

### Metagenomic read recruitment onto genome catalogs

We assessed the fraction of metagenomic reads recruited onto the OceanDNA MAGs. Sequence reads of the 2,057 metagenomes used for genome reconstruction were back mapped onto the 8,466 species representatives of the OceanDNA MAGs. If multiple sequencing runs were performed for one sample, only a run of the largest scale was used. Read mapping was performed with bowtie2^38^ v2.3.5.1 with the default setting using the quality-controlled paired-end reads of each run. If it is the case that the run was larger than 5 Gbps, a subset of 5 Gbps were randomly sampled using seqtk (https://github.com/lh3/seqtk) v1.3 and used for the read mapping. Then, the mapping result was sorted using samtools (http://www.htslib.org/) v1.9, and mappings of ≥95% identity, ≥80 bp, and ≥80% aligned fraction of the read length were extracted using msamtools (https://github.com/arumugamlab/msamtools) that are bundled in MOCAT2^56^ v2.1.3. Finally, the mapped reads were counted using featureCounts^68^ bundled in Subread v2.0.0. The species representatives collectively cover 10.4–35.0% (the first and third quartiles) of metagenome reads of the 2,057 metagenomes (Fig. 3a). Especially where only prokaryotes-enriched metagenomes (n = 731) were considered, 26.5–42.0% of metagenomic reads were mapped onto the species representatives.

Next, we evaluated mapped read fractions onto species representatives of UGCMP, the OceanDNA MAGs, and the other genome sets of marine prokaryotic genomes from large-scale genome reconstruction studies^3,5,16,66^ (Fig. 3b). Read mapping was performed using only species representatives of qualified genomes (i.e., QS ≥ 50) for all these genome collections. Regarding the medians of mapped read fractions, the OceanDNA MAGs were the highest (34.6%) among the previously reported genome collections, and UGCMP (43.4%) was 9.2% higher than the OceanDNA MAGs.

## Data Records

Genome sequences of the OceanDNA MAGs were available at figshare^52^ and submitted to DDBJ/ENA/GenBank under BioProject accession no. PRJDB11811^53^. Genome sequences of the 8,466 species representatives were submitted as WGS entries under BioProject accession no. PRJDB11811^53^, and available at figshare^52^. Genome sequences of non-representatives (n = 43,859) were submitted as DDBJ analysis entries^69^ (available only via DDBJ) and available at figshare^52^. Supplementary files are also available at figshare^52^.

## Technical Validation

For maximization of the genome quality, our genome reconstruction pipeline was carefully designed, including three key processes (Fig. 1c):

1. High-resolution coverage profiles were calculated using all metagenomes within each division.
2. Metagenome binning was performed using three algorithms and subsequently dereplicated.
3. An automated post-refinement process was developed to detect possible contaminations, including ones that are likely missed by prokaryotic single-copy marker gene-based assessment.

Here we assessed the effectiveness of these processes.

**Fig. 4:**
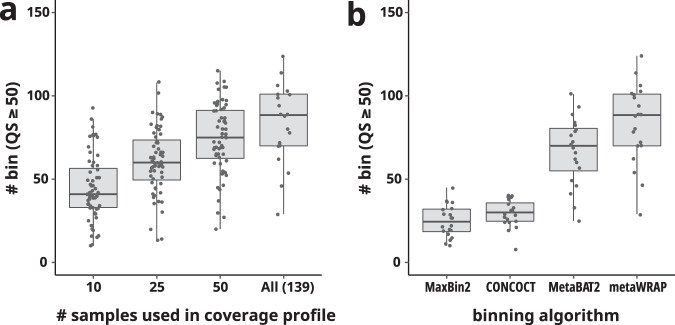
Assessment of the genome reconstruction pipeline. Using selected 20 *Tara* Oceans metagenomes included in the “*Tara* prok” division, the impact of high-resolution coverage profiles (**a**) and the use of multiple binning algorithms (**b**) were assessed. The number of qualified genome bins (QS ≥ 50) was compared between (**a**) coverage profiles calculated with all metagenomes within the same division (n = 139) or with randomly sampled 10, 25, and 50 metagenomes (3 replicates), and between (**b**) different algorithms: MaxBin2, CONCOCT, MetaBAT2, and merged results of the three algorithms using the bin_refinement module of MetaWRAP.

First, binning algorithms primarily depend on a coverage profile among multiple metagenomes and *k*-mer (e.g., tetranucleotide) composition of metagenomic contigs^70,71^. If a coverage profile was calculated using only a few metagenomes, it would underperform a binning algorithm (e.g., CONCOCT^41^). Here, to assess the effect of the number of metagenomes in a coverage profile, we selected 20 *Tara* Oceans metagenomes included in the “*Tara* prok” division (Table 1), of which geographic region and water depth were widely distributed. We performed metagenome binning of the selected metagenomes with different coverage profiles. The coverage profiles were calculated with all metagenomes within the same division (n = 139) or randomly sampled 10, 25, and 50 metagenomes with three replicates out of the 139 metagenomes. If multiple sequencing runs were available from one metagenome, a run that produced the largest amount of sequence was used for coverage profiles. Then, binning was performed in the same way as the OceanDNA MAGs, except for the post-refinement part, and the resulting number of bins of QS ≥ 50 was compared (Fig. 4a). As a result, coverage profiles of all metagenomes reconstructed the greater number of qualified bins (i.e., QS ≥ 50) than coverage profiles of subsampled metagenomes. The result suggests the superiority of the ‘high-resolution’ coverage profiles incorporating more metagenomes.

Second, using the same 20 metagenomes of the “*Tara* prok” division, the binning result of a single algorithm (MetaBAT2, CONCOCT, MaxBin2) and the dereplicated result of the three algorithms using the bin_refinement module of MetaWRAP were compared (Fig. 4b). Dereplication of bins generated from three algorithms significantly increased the number of qualified bins (i.e., bins of QS ≥ 50).

Third, we designed an automated post-refinement process using three filters that are independent of prokaryotic single-copy marker genes: (1) taxonomic filter, (2) mobile element filter, and (3) outlier filter. Similar strategies were applied in previous studies (e.g., MAGpurify^72^, GUNC^73^). This refinement process aims to remove contamination for genome quality improvement. Especially, contamination over the domain (i.e., eukaryotic and viral contigs included in prokaryotic genomes) would not be detected through analysis of prokaryotic single-copy marker genes. For example, several genomes reported from *Tara* Oceans MAG studies were predicted to contain many viral contigs (in a few cases, more than 50) within a single genome^74^. Viral contigs are possible contaminants with similar coverage profiles and *k*-mer compositions to the prokaryotic genome^22^. Though the removal of viral and plasmid sequences possibly results in the exclusion of an actual element of the genome (e.g., provirus and plasmid as a part of the genome) and identification of viral and plasmid contigs might contain false positives, we placed a high priority on removing those as possible contamination for better genome quality.

The three filters of the post-refinement module identified 561,804, 39,289, and 436,143 potential misassigned contigs, respectively. Overall, from 54,614 qualified genome bins, 1,000,417 contigs were filtered out (18.3 contigs per genome bin on average), and 2,289 genome bins were discarded due to the reduction of genome completeness (i.e., the QS drops below 50) caused by the decontamination process. Code for the post-refinement process is available at GitHub as a tool named MAGRE (https://github.com/yosuken/MAGRE).

## Usage Notes

We collected metagenome data covering various marine environments for the large-scale reconstruction of marine prokaryotic genomes. The metagenome dataset was primarily focused on water samples, and sediment trap and biofilm samples were also included. It should be noted that some marine environments (e.g., sediments, hydrothermal vents, and coral reefs) were not included in the dataset.

We carefully designed the genome reconstruction pipeline for genome quality improvement, including the automated post-refinement process. Nevertheless, due to the difficulty of perfect decontamination, misassigned contigs might still be included in the genomes. Manual quality control is recommended before the use of the genomes, as is the case for MAGs reported from other studies.

Genome completeness evaluated by CheckM is likely underestimated for genomes of specific taxa that have experienced extreme genome reduction and may have a symbiotic lifestyle (e.g., lineages of the phylum Patescibacteria, also known as the Candidate Phyla Radiation). Ribosomal RNA operons are challenging genomic regions to reconstruct due to the co-existence of closely related sequences that confuse de Bruijn graph-based assemblers^22^. 5 S, 16S, 23 S ribosomal RNAs were identified in 24.2%, 6.8%, 3.8% of the OceanDNA MAGs, respectively (including complete sequences and >25% fragments of the whole length). We assigned quality tiers according to the MIMAG standard^75^ (Supplementary File S3). Due to the difficulty of reconstructing ribosomal RNA operons, only 108 genomes were assigned to the high-quality drafts, and the remaining genomes (n = 52,217) were the medium-quality drafts.

The fraction of mapped reads onto the OceanDNA MAGs was not high, even for prokaryote-enriched metagenomes (Fig. 3a; 26.5–42.0%, the first to third quartiles). We consider there are at least threefold reasons. First, the mapping was limited to the species representatives, and the mapping criteria were stringent (i.e., ≥95% nucleotide identity). The inclusion of non-representatives or the use of a more relaxed threshold would result in a larger fraction of mapped reads. If we changed the mapping criteria to ≥90% nucleotide identity, the mapped fraction was increased by ~7% (34.2–49.6%, the first to third quartiles). A similar case was reported from a marine SAG study^5^, which showed that the nucleotide identity threshold significantly affected the fraction of mapped reads onto a genome collection.

Second, marine metagenomes possibly include a substantial fraction of viruses and eukaryotes, even in prokaryote-enriched metagenomes. We performed a domain-level assignment of metagenomic reads using Kaiju^76^ v1.8.2 with NCBI nr as a reference database. The domain-level classification of prokaryote-enriched metagenomes showed that the majority were prokaryotic reads (51.5%–62.1%, the first to third quartiles; Supplementary File S1). Although the fraction of viral and eukaryotic reads was small as a general trend (0.39%–1.66% for eukaryotes and 0.56%–1.79% for viruses), some prokaryote-enriched metagenomes include substantial fractions of eukaryotic (up to 9.88%) or viral reads (up to 34.1%). Furthermore, considering the fraction of ‘unclassified’ reads being large (35.5%–45.6%) and the lack of reference genomes of marine eukaryotes and viruses in the database, the fraction of viruses and eukaryotes is considered underestimated.

Third, the SAR11 clade and the genus *Prochlorococcus* are abundant prokaryotic lineages in the ocean. However, despite their expected high abundance, a relatively small number of genomes were reconstructed in this study. This shortage is attributable to coexisting closely related strains of these lineages that confuse de Bruijn graph-based assemblers^22^. Among the OceanDNA MAGs, 780 genomes were reconstructed from 85 species-level clusters of ‘o__Pelagibacterales’ (SAR11), and 157 genomes were reconstructed from 8 species-level clusters of ‘g__Prochlorococcus’, according to the GTDB classification. For these lineages, SAGs could supplement genomic information. For example, recently reported SAGs that were reconstructed from the tropical and subtropical euphotic ocean^5^ includes 2,108 genomes consisting of 1,215 species-level clusters of ‘o__Pelagibacterales’ and 327 genomes consisting of 155 species-level clusters of ‘g__Prochlorococcus,’ where genomes are limited to those of QS ≥50 (Supplementary File S5).

## Supplementary information

A list of supplementary files

Supplementary File S1

Supplementary File S3

Supplementary File S4

Supplementary File S5

Supplementary File S6

## Data Availability

Code of the post-refinement module, named MAGRE, is available at GitHub (https://github.com/yosuken/MAGRE). The options and parameters of all tools used for the analysis are described in the main text.
